# Fluoroscopic‐assisted laparoscopic retrieval of retained glucose sensor wire from the omentum

**DOI:** 10.1002/ccr3.2348

**Published:** 2019-08-04

**Authors:** Adam X. Sang, Rayhan Lal, Auriel August, Enrico Danzer, Bruce Buckingham, Claudia M. Mueller

**Affiliations:** ^1^ Division of Pediatric Surgery, Department of Surgery Stanford University Stanford California; ^2^ Division of Endocrinology, Department of Medicine & Department of Pediatrics Stanford University School of Medicine Stanford California

**Keywords:** children, continuous glucose monitoring, retained foreign body, type 1 diabetes

## Abstract

We describe a case in which retained wires from a continuous glucose monitor were removed from the abdominal wall and peritoneum of a 6‐year‐old boy. We highlight a concern for continuous glucose monitor use in children and discuss surgical techniques used to retrieve tiny, mobile objects from complex body cavities.

## 1 INTRODUCTION

Retention of a foreign body is a known complication of many medical devices. Retained foreign bodies are frequently discovered incidentally, and asymptomatic foreign bodies can oftentimes be managed nonoperatively. However, certain foreign bodies have the potential to migrate, and may injure tissues and organs, prompting immediate surgical retrieval.

Continuous glucose monitors (CGMs) are increasingly popular devices for patients with type 1 diabetes mellitus. A hair‐thin wire is introduced over a needle subcutaneously, usually into the abdominal wall, to facilitate real‐time reading of glucose levels from the interstitial fluids in the subcutaneous tissue as a proxy for blood glucose. The accidental introduction of this sensor wire into the peritoneal cavity is a theoretical risk of this technology.^1^

We present the case of a child with two retained wires from a CGM system, including one unexpectedly trapped inside the peritoneal cavity, to highlight this rare problem and illustrate intraoperative strategies for finding and extracting such a small, mobile object.

## 2 CASE REPORT

**Figure 1:**
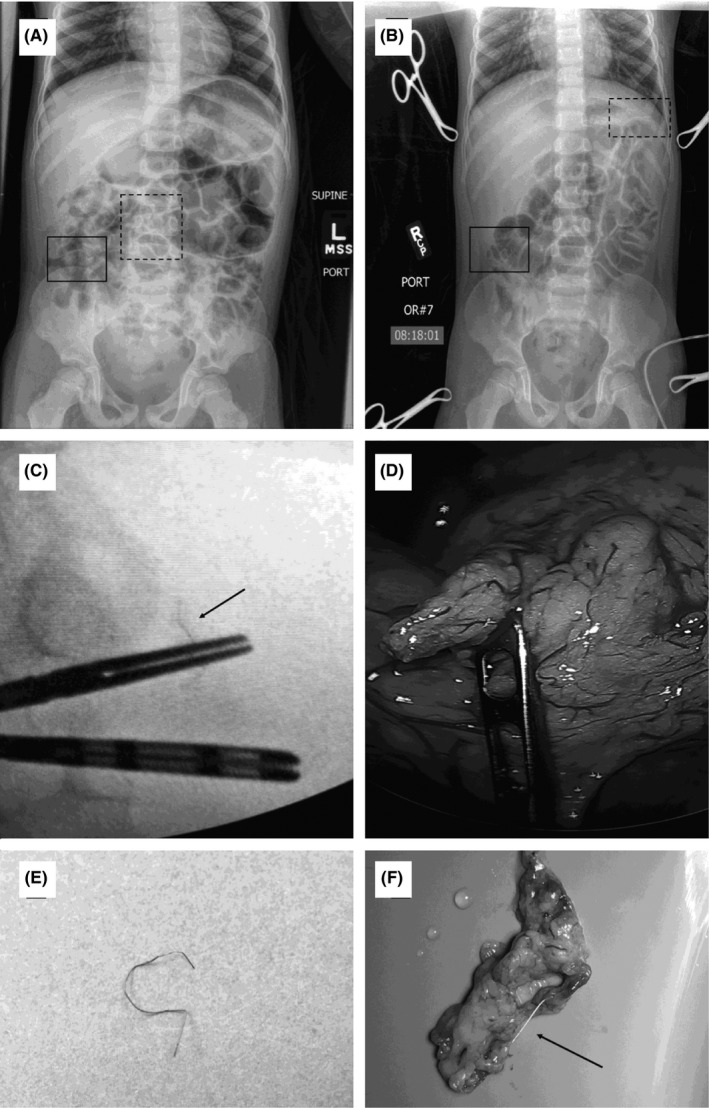
Surgical retrieval of retained glucose sensor wire. A, Preoperative abdominal X‐ray shows the location of the two retained wires (solid box and dotted box). B, Intraoperative abdominal X‐ray demonstrates that one of the wires (dotted box) had migrated to the left upper quadrant in the interim. The location of the other wire was unchanged and was uneventfully retrieved from the abdominal wall. C, Under fluoroscopy, blunt graspers were used to localize and clamp the intra‐abdominal wire (arrow). D, Using laparoscopy, the tissue containing the migrating intra‐abdominal wire was found to be the omentum. E, The retrieved wire from the abdominal wall (the wire in the solid box). F, The retrieved wire from the omentum (the wire in the dotted box)

The patient is a lean 6‐year‐old boy with a history of celiac disease who was diagnosed with type 1 diabetes mellitus and started on a continuous glucose monitor. Five months later, his parents placed a new sensor in the right lower quadrant abdominal wall. They did not receive a signal from the sensor and removed it, but noticed the wire had detached. By the following week, pain, swelling, and redness were noted over the site. An X‐ray revealed the retained wire within the abdominal wall at the insertion site. An elective removal of the wire under sedation was scheduled with pediatric surgery. Prior to removal, the parents reported placing another sensor from the same box into the abdominal wall in the left lower quadrant. Once again, they did not receive a signal from the sensor, removed it, and found that the wire had detached. Another X‐ray was obtained which showed a foreign body on the right side, corresponding to the first lost wire (Figure 1A, solid box), and a second wire in the midline, quite distant from where the parents had initially inserted it (Figure 1A, dotted box).

At the time of surgery, the first wire was easily palpable within the subcutaneous tissue of the right lower quadrant abdominal wall and was removed via a small skin incision (Figure 1E). The second wire was not palpable, and an X‐ray taken on the operating room table showed that the wire had migrated from the midline to the left upper quadrant (Figure 1B, dotted box).

Having concluded from these images that the second wire was likely within the peritoneal cavity, we performed a diagnostic laparoscopy. We were unable to locate the wire with direct inspection. With the aid of intraoperative fluoroscopy, the wire was ultimately found to be embedded within the omental tissue (Figure 1C and 1D). The piece of omentum encasing the wire was removed with electrocautery. The specimen was inspected grossly, and the wire was identified (Figure 1F). A postretrieval X‐ray demonstrated successful removal of both wires.

**Figure 2:**
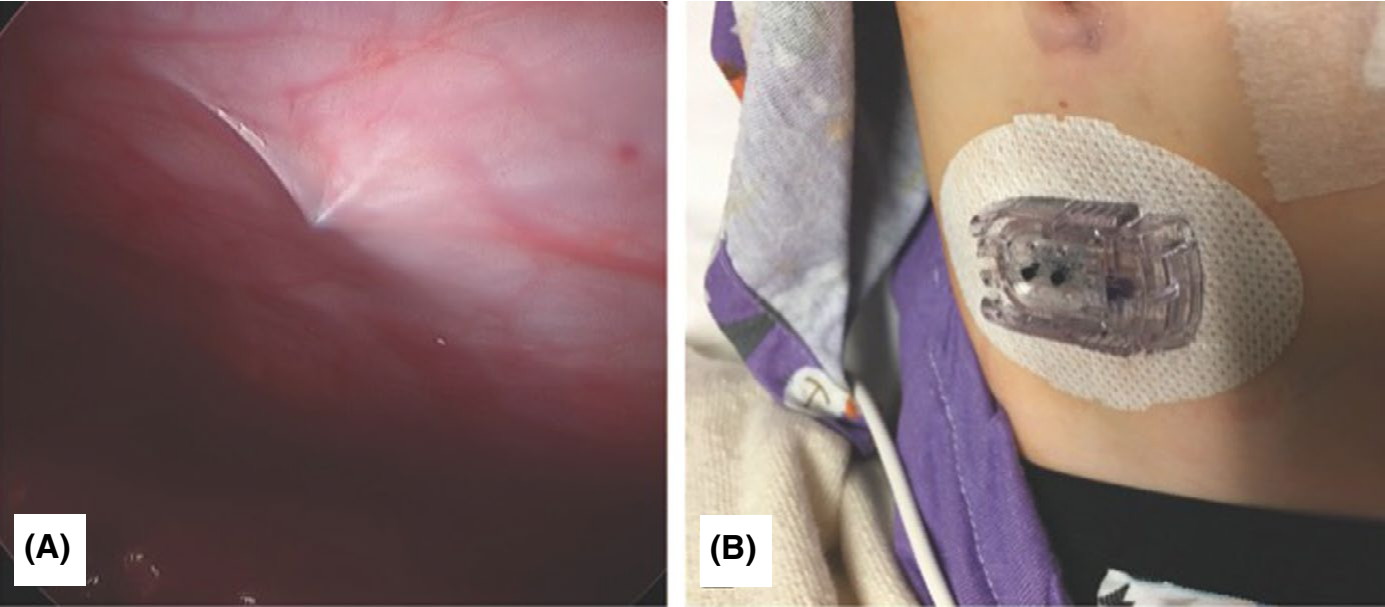
Placement of a new glucose sensor wire under laparoscopic visualization. A, Placement of the device on the abdominal wall and deployment of the sensor wire per manufacturer's instructions resulted in peritoneal penetration. B, External view of the monitoring device after the sensor wire was deployed obliquely in the abdominal wall without peritoneal penetration

Finally, under direct laparoscopic visualization, we placed a new sensor percutaneously per instruction. The needle did indeed penetrate the peritoneum on initial insertion (Figure 2A). We then adjusted the angle of deployment of the needle so that we could place it without violating the peritoneum (Figure 2B). The findings were shared with the parents, our colleagues in endocrinology, and the manufacturer of the device. The patient was admitted overnight for monitoring and discharged the next day. He is currently using the same continuous glucose monitor system to help him maintain glycemic control.

## 3 DISCUSSION

Continuous glucose monitoring is becoming an increasingly common standard of care for children with type 1 diabetes. It is an appealing technology for its general ease of use. In addition, there is clear evidence that when patients use continuous glucose monitors, glycemic control is improved compared with traditional capillary blood sugar.^2^, ^3^

Given the increased popularity of these devices, clinicians should be aware of the rare risk of peritoneal needle introduction and foreign‐body retention. While sensor wires dislodged into the subcutaneous tissue might be a nuisance, they are generally benign and relatively easy to locate due to their static nature. By contrast, once the wires penetrate the peritoneum, they become freely mobile and can cause more serious consequences. Interestingly, insertion into the peritoneum may have implications for sensor kinetics, as a previous study demonstrated a reduction in mean time constant from 12.4 minutes in the subcutaneous space to 5.6 minutes in the intraperitoneal cavity in eight swine models.^4^

To our knowledge, this is the first description of retrieving percutaneously introduced glucose sensor wires from the peritoneal cavity. With regard to other diabetes technology, there are reports of subcutaneous (not intraperitoneal) needle detachment from steel insulin infusion sets, removed uneventfully under fluoroscopy.^5^, ^6^ Most foreign objects retrieved from the abdominal cavity arrive there after ingestion and transluminal penetration.^7^, ^8^, ^9^ These were almost always removed with laparoscopy or endoscopy. Intraoperative fluoroscopy has been used to assist with localization and compensate for the limited tactile feedback with laparoscopy.^7^

In our case, the intraperitoneal wire retrieval was challenging for several reasons. First, the size of the target object was subcentimeter and very thin. In addition, the object migrated continuously during our search, as we grasped or retracted different tissues without knowing the exact location of the target. Finally, the operative field (abdominal cavity) was complex and heterogeneous. We began by directly inspecting the peritoneal cavity contents with the laparoscope and a grasper in the general region of where we suspected the wire to be, based on static X‐ray images. This approach was unsuccessful, in part due to the fact that the wire kept changing locations from image to image, and we were always behind on the chase.

The use of live fluoroscopy allowed us to eventually align the jaws of the laparoscopic grasper over the opaque wire. The surgeon accomplished this by looking just at the fluoroscopy monitor and not the laparoscopy screen. Afterward, attention was turned back to the laparoscopy screen, and the tissue immediately under the grasper was retracted upward. In this case, it was the omentum. The final key maneuver was another three seconds of live fluoroscopy, during which the surgical team jiggled the grasper (presumably holding the foreign body) and simultaneously observed both screens to ensure that the grasped object moved in concert under both live fluoroscopy and laparoscopy (Figure 1C and 1D).

Overall, retained wires are a relatively uncommon phenomenon among thousands of users who wear sensors. As reported in 2011, it is believed to occur in 0.03% of sensors shipped.^8^ Furthermore, the clinical consequences of wire retention are generally benign, especially in an asymptomatic patient. However, device deployment is ideally performed where there is sufficient subcutaneous tissue to pinch during placement. Pediatric patients have a choice between two labeled wear sites: the abdomen and upper buttock. Younger and thinner patients may be at greater risk of needle and wire penetration deep to the abdominal wall and into the peritoneal cavity. Thus, the upper buttock site may be a better option for these patients.

In retrospect, the fact that an abdominal X‐ray, which was taken in the emergency room, showed the second wire on the contralateral side from its insertion site might have served as a clue to its mobility. During the case, we could also directly see the needle enter the peritoneum during laparoscopy (Figure 2A). This suggests that oblique insertion might be less likely to result in needle and wire penetration of the peritoneal cavity. Further, a future sensor with a shorter wire may be better suited for pediatric patients. We hope this case alerts providers and patients to minimize the risk of both wire retention and introducer needle penetration into the peritoneum.

## CONFLICT OF INTERESTS

Dr Lal has consulted for GlySens Incorporated and Abbott Diabetes Care. Dr Buckingham has received research support for PI‐initiated studies as well as sensors at a research discount for closed loop studies from Dexcom.

## AUTHOR CONTRIBUTIONS

Adam Sang: wrote and edited the manuscript; Rayhan Lal and Auriel August: edited and reviewed the manuscript; Enrico Danzer and Bruce Buckingham: revised the manuscript; and Claudia Mueller: conceived the case report, and edited and revised the manuscript.
